# Impact of Support (MCF, ZrO_2_, ZSM-5) on the Efficiency of Ni Catalyst in High-Temperature Conversion of Lignocellulosic Biomass to Hydrogen-Rich Gas

**DOI:** 10.3390/ma12223792

**Published:** 2019-11-19

**Authors:** Jacek Grams, Robert Ryczkowski, Karolina Chałupka, Izabela Sobczak, Izabela Rzeźnicka, Kamila Przybysz

**Affiliations:** 1 Institute of General and Ecological Chemistry, Faculty of Chemistry, Lodz University of Technology, Zeromskiego 116, 90-924 Lodz, Poland; robert.ryczkowski@gmail.com (R.R.); karolina.chalupka@p.lodz.pl (K.C.); 2 Adam Mickiewicz University, Faculty of Chemistry, Uniwersytetu Poznańskiego 8, 61-614 Poznan, Poland; sobiza@amu.edu.pl; 3 Graduate School of Science and Engineering, Shibaura Institute of Technology, 135-8548 Tokyo, Japan; izabela@shibaura-it.ac.jp; 4 Natural Fibers Advanced Technologies, Blekitna 42A, 93-322 Lodz, Poland; kamila.przybysz@interia.pl

**Keywords:** nickel catalyst, hydrogen-rich gas, lignocellulosic biomass, cellulose, pyrolysis, MCF, zeolite, ZrO_2_, thermal decomposition

## Abstract

The main objective of this work was to evaluate an impact of a support on the efficiency of nickel catalysts in the high-temperature conversion of lignocellulosic biomass to hydrogen-rich gas. The most important parameters influencing catalytic performance of the catalysts were identified. The properties of three materials (ZSM-5, ZrO_2_, and MCF (mesostructured cellular foam)) used as a support differing in surface acidity, surface area, pore structure, ability to interact with an active phase, and resistance to coking, have been studied. The results revealed that Ni/MCF, characterized by large pore size and pore volume, low acidity, small NiO crystallites size, and moderate interaction with the active phase, is the most efficient among studied catalysts, while an application of Ni on ZSM-5 support with high-acidity was not beneficial. The results suggest that structure of the support, in particular larger pore size and a better contact between an active phase and reaction intermediates, play an important role in the formation of gaseous products during thermal decomposition of lignocellulosic feedstock. On the other hand, high acidity of the support did not increase the formation of large amounts of hydrogen-rich gaseous products.

## 1. Introduction

In recent years, lignocellulosic biomass has become one of the most popular renewable sources of energy and fine chemicals. This fact results from its global availability, relatively low price, and limited influence on the increase in the carbon dioxide emission. This type of feedstock is often processed with the use of a high-temperature treatment leading to the formation of gaseous products, liquid fraction (bio-oils), and solid residues [1,2]. One of the most valuable gaseous products present in the gas mixture is hydrogen. Currently, hydrogen gas is mainly produced via steam reforming of natural gas or coal gasification. The production of hydrogen from lignocellulosic biomass is an attractive alternative from the environmental point of view. However, the efficiency of this process is not satisfactory due to the complex mechanism and formation of numerous by-products [3,4]. The effectiveness of the lignocellulosic biomass conversion can be considerably improved by use of heterogeneous catalysts [5,6]. Literature reports indicate that nickel-based materials are one of the most efficient catalysts used in high-temperature conversion of lignocellulosic biomass [7,8]. However, the activity of Ni catalysts strictly depends on the support onto which Ni was dispersed [9].

Moghtaderi [10] applied Ni/Al_2_O_3_, prepared by the co-precipitation method, for the steam gasification of pine sawdust towards the production of hydrogen. The results revealed that nickel catalyst increased selectivity of H_2_ production. The catalyst with NiO:Al_2_O_3_ = 50:50 molar ratio was the most efficient in the reduction of tar content and formation of gaseous products, while the materials with lower NiO contribution (20 mol.% or 25 mol.%) were less effective. On the other hand, Li et al. [11] reported the use of Ni/Al_2_O_3_, prepared by the deposition precipitation method, for hydrogen production via air-steam gasification of a rice husk. In this case, the content of the introduced nickel oxide was about 12 wt.%. The catalyst facilitated cracking of tar presented in the gasification products and increased the efficiency of reforming and water gas-shift reactions. Its catalytic activity was most likely associated with a better dispersion of an active phase and high surface area of above 120 m^2^/g.

However, Ni catalysts supported on Al_2_O_3_ may suffer from deactivation due to carbon deposit formation [12]. Therefore, researchers are focusing on the studies of other types of materials. Lu et al. [13] investigated the catalytic performance of 20 wt.% Ni/Al_2_O_3_ and 20 wt.% Ni deposited on 5 wt.% CeO_2_-Al_2_O_3_ for hydrogen production by gasification of glucose as a model biomass sample. It was demonstrated that an addition of ceria allowed for a slight increase in the selectivity and hydrogen yield. The high efficiency of the catalyst was attributed to the higher resistance of Ni/CeO_2_-Al_2_O_3_ against carbon deposition (additionally, more filamentous coke was formed instead of a graphitic one) and the hindering of the growth of nickel species on the support surface during the reaction. The same was observed by Tomishige et al. [14] during steam gasification of cedar wood. The authors suggested that the enhancement of catalyst activity and hydrogen production was related to the formation of a strong interaction between nickel and ceria.

The addition of ceria also appeared to be beneficial in the case of a 15 wt.% Ni/CeO_2_-ZrO_2_ system, tested in the steam reforming of tar formed in benzene (used as a model compound) gasification [15]. The Ni/CeO_2_-ZrO_2_ catalyst possessed enhanced redox properties and resistance against coke deposition. Same conclusions were obtained in our previous work devoted to investigations of Ni/CeO_2_-ZrO_2_ catalyst in the high-temperature conversion of cellulose [16]. Our results suggested that the reduction of catalyst deactivation rate can be related to the presence of Ce^3+^ species on the surface of the catalyst.

Although, catalytic systems described above showed promising properties, some researchers investigated the potential of monoxide supports. Kong et al. [17] studied the effect of Al_2_O_3_, SiO_2_, ZrO_2_, and MgO supports of Ni catalyst for CO_2_ reforming of toluene, used as a model compound of biomass gasification tar. The results demonstrated that the type of oxide influences both the distribution and the stability of Ni active phase. Ni supported on MgO showed the highest activity, which was attributed to the basic character of magnesium oxide, higher dispersion of Ni species on the surface of the catalyst, and formation of Ni-Mg-O solid solution. To the contrary, in the studies described in Reference [18], the most promising support of a Ni catalyst for high-temperature conversion of biomass/cellulose conducted in the absence of oxygen was ZrO_2_. Its catalytic performance was strongly dependent on the preparation method, crystallographic phase, and thermal stability, among others [19,20].

Zeolites or mesoporous silicas have also been tested as supports for Ni catalysts in high-temperature conversion of a biomass. Zeolites having a higher acidity facilitate cracking of lignocellulosic feedstock, but simultaneously undergo more intense coking [21]. French and Czernik [22] reported that during catalytic pyrolysis of biomass, ZSM-5-based materials showed higher deoxygenation activity in comparison to zeolites having larger-pores. On the other hand, literature reports show examples of Ni catalysts supported on mesoporous silicas (MCM-41, KIT-6, SBA-15) used for upgrading the composition of products obtained in the thermal conversion of lignocellulosic biomass [23,24,25]. In spite of the lower acidity of these materials, mesopores can facilitate decomposition of more complex intermediates of the pyrolysis process due to larger pore size and enhanced penetration.

As described above, the problem of the support selection for a nickel catalyst has been raised in literature reports. However, an interpretation of the results is not unequivocal, especially when it comes to the factors that determine the activity of the prepared catalysts to the greatest extent, such as acidity, porosity, stability, interaction with the active phase, etc. Therefore, in this work, we compared the properties of three different materials that could be used as a support of Ni catalyst in the high-temperature conversion of lignocellulosic biomass to hydrogen-rich gas and indicated the most important parameters influencing their catalytic behavior in the process. Based on literature research and our previous reports, we employed ZSM-5, ZrO_2_, and MCF (mesostructured cellular foam). ZSM-5 is an aluminosilicate zeolite possessing a high surface acidity, ordered structure, and relatively small pores [26]. MCF is a type of mesostructured cellular foam silica consisting of large spherical cells, which are connected by uniformly sized windows, giving a three-dimensional (3D) pore system with a larger pore size than in the case of zeolites [27]. ZrO_2_ exhibits lower surface area in comparison to silica-based materials. However, it can interact with the active phase of the catalyst more strongly and exhibits a higher resistance against carbon deposition [28].

## 2. Experimental

### 2.1. Preparation of Ni Catalyst Supports

ZrO_2_ was obtained from ZrOCl_2_∙8H_2_O (Sigma–Aldrich) by precipitation with NaOH followed by calcination at 700 °C in air. First, 200 mL of aqueous solution of 0.4 M ZrOCl_2_∙8H_2_O was added dropwise to 60 mL of aqueous solution of 5 M NaOH (StanLab, Lubin, Poland). Afterwards, the mixture was heated to 104 °C and stirred for 24 h. The precipitate was filtered on a Büchner funnel and washed with 0.05 M aqueous solution of NH_4_NO_3_ (Chempur, Piekary Slaskie, Poland) and distilled water until neutral pH. Subsequently, it was dried in air at 110 °C overnight and calcined in air at 700 °C for 4 h (with the temperature ramp of 1 °C/min from a room temperature to 400 °C, and 20 °C/min temperature ramp to reach 700 °C) to obtain ZrO_2_.

MCF was prepared by a one-pot synthesis method according to the procedures given in Reference [29]. First, Pluronic 123 (poly(ethylene glycol)-block-poly(propylene glycol)-block-poly(ethylene glycol)-block) (8 g, 1.4 mmol) was dissolved in 300 g of 1.6 M HCl solution at 35–40 °C. Next, 1, 3, 5-trimethylbenzene (Aldrich) (8 g) and NH_4_F (Aldrich) (0.0934 g, 2.52 mmol) were added under vigorous stirring. Following 1 h of stirring, tetraethyl orthosilicate, TEOS (Fluka) (17.054 g, 81.99 mmol) was added. The final mixture was stirred at 35–40 °C for 24 h and then transferred into a polypropylene bottle and heated at 100 °C under static conditions for 24 h. The solid product was recovered by filtration, washed with distilled water, and dried at room temperature. The template was removed from the as-synthesized material by calcination at 500 °C for 8 h under static conditions.

A commercial ZSM-5 (Merck) was used as a base for the investigated support. The material was calcined in the air flow in 500 °C for 4 h in order to obtain H-ZSM-5.

### 2.2. Preparation of Ni Catalysts

The supported 20% Ni catalysts (about 5 g) were prepared by the impregnation method. Nickel was introduced from an aqueous solution of Ni(NO_3_)_2_·6H_2_O (Chemland, Stargard Szczecinski, Poland, pure for analysis (≥99.5%)) on the surface of ZSM-5, ZrO_2_, and MCF supports, prepared earlier. The samples were aged for 24 h at room temperature. After evaporation of water, the catalysts were dried at 120 °C for 2 h, and then calcined in air flow at 700 °C for 4 h (with the temperature ramp of 1 °C/min from room temperature to 400 °C, and 20 °C/min to reach 700 °C) to allow for a slow nitrate precursor decomposition. Prepared catalysts were stored in sealed containers before activity measurements.

### 2.3. Characterization of the Catalysts

The surface area measurements were conducted on an ASAP2010 Micromeritics (Norcross, United States of America) using N_2_ as an adsorbent at −196 °C, with a prior outgassing at 200 °C for 3 h in order to desorb any impurities or moisture. The Brunauer-Emmett-Teller (BET) specific surface area was calculated from the N_2_ adsorption isotherm. Pore radius and pore volume of Ni/ZrO_2_ and Ni/ZSM-5 catalysts were determined by the Barrett, Joyner, and Halenda (BJH) method. The pore volume and diameter of Ni/MCF was estimated from adsorption branches of N_2_ isotherms according to the Broekhoff-de Boer method with the Frenkel-Halsey-Hills approximation [30].

Powder X-ray diffractograms (XRD) were collected using a PANalytical X’Pert Pro MPD diffractometer (Malvern, UK). The X-ray source was a copper, long, fine focused X-ray diffraction tube operating at 40 kV and 30 mA. Data were collected in the 5–90° 2*θ* range with 0.0167° step. Crystalline phases were identified by references to ICDD PDF-2 (Version 2004) database. All calculations were performed with the X’Pert HighScorePlus computer program (Version 2004, Malvern Panalytical, Malvern, UK). The calculation of NiO crystallite size was based on the Scherrer equation.

Temperature-programmed reduction (TPR) was performed on the AMI1 system from Altamira Instruments equipped with a thermal conductivity detector (TCD) and used for examining the reducibility of the catalysts. In the experiments, a mixture of 5 vol.% H_2_ and 95 vol.% Ar was applied with the flow rate of 30 mL/min and a linear temperature ramp of 10 °C/min.

Acidity of the measured samples was determined by temperature-programmed desorption of ammonia (TPD-NH_3_) in a dynamic mode. Prior to the measurements, each sample was heated at 500 °C for 1 h in an argon atmosphere. After cooling the samples to 100 °C, adsorption of ammonia was performed for 15 min. Afterwards, flushing of the samples with argon was carried out for 15 min. The measurements were performed by heating the samples to 500 °C with the ramp rate of about 25 °C/min and registering the amount of desorbed ammonia using TCD.

Scanning electron microscope (SEM) Jeol FE-SEM 7100F (Tokyo, Japan), equipped with an energy-dispersive X-ray (EDX) detector, was used for the investigation of the morphology and composition of the catalysts. The measurements were taken at the acceleration voltage of 15.0 kV.

Thermogravimetric analysis/differential thermal analysis/mass spectrometry (TGA/DTA/MS) was performed using derivatograph SETSYS 16/18 (Caluire, France), Setaram and a mass spectrometer ThermoStar, Balzers (Asslar, Germany). The TGA-DTA and MS spectra were recorded in the air flow (40 cm^3^/min) in the temperature range of 10–900 °C with the heating rate of 10 °C/min. The sample mass was between 5 and 20 mg. All the samples were weighted in the corundum crucible. These studies were used to determine the content and reactivity of carbon deposits.

### 2.4. Catalytic Activity Tests

The activity of the prepared nickel catalysts was tested in a two-step, fixed bed quartz reactor (with separated biomass and catalytic bed) close to the atmospheric pressure. The minimal flow of Ar was used in order to direct produced gases from the reactor to the gas chromatograph. The total gas flow was maintained at 15 mL/min. The catalyst bed was held at 700 °C and cellulose/biomass was heated to 500 °C. Obtained products were directed to the catalyst bed where they underwent subsequent upgrading. In the next step, an evaluation of the total gas volume was performed. Subsequently, the obtained mixture was passed to the gas chromatograph equipped with TDC. The amounts of H_2_, CO, CO_2_, and CH_4_ were determined in this way. The reaction temperature was chosen based on the preliminary tests.

Model biomass sample, an α-cellulose (Sigma–Aldrich, Saint Louis, MO, USA, pure), and a real biomass pine pulp (consisting of 94.5% of cellulose, 2.5% of hemicellulose, and 2.6% of lignin) were used in the catalytic tests. In each test, 0.4 g of a feedstock and 0.1 g of the catalyst were used.

Pine cellulosic pulp was prepared by the Kraft method from air-dried woodchips (25 mm × 15 mm × 18 mm). Dry weight (d.w.) of all materials was determined before pulping. The delignification process was conducted in a 15 L stainless-steel (ANSI 316 L) reactor with temperature control (PD-114, Danex, Katowice, Poland) and agitation system (3 swings per minute, swinging angle of 60°). Suspensions of woodchips (1000 g d.w., liquid modulus equal 4, active alkali 38%, sulfidity 30%) were heated for 120 min to achieve a temperature of 172 °C, and incubated at this temperature for the next 120 min. Then, the temperature was decreased to ambient temperature (22 °C) using a jacket with cold tap water. Insoluble residues were separated by the filtration within the reactor, washed with approximately 80 L of demineralized water, and incubated overnight (12 h) in demineralized water to remove residues of the alkali-soluble fractions. The obtained fibrous biomass was disintegrated using a laboratory JAC SHPD28D propeller pulp disintegrator (Danex, Katowice, Poland) at 10,000 revolutions and screened using a PS-114 membrane screener (Danex, Katowice, Poland) (0.2 mm gap). Fibers were collected, dried for 48 h in ambient conditions (21 °C), and then weighed.

## 3. Results

### 3.1. Catalytic Activity

An analysis of gaseous products formed in the high-temperature conversion of cellulose and pine showed that the mixture mainly consists of hydrogen, carbon oxide, carbon dioxide, and methane (Table 1 and Table 2). The volume of produced gases and their contribution in the reaction mixture strictly depended on the presence of the catalyst and type of support being used.

The lowest amount of gaseous products was formed in the case of non-catalytic process and in the presence of the pure supports (220–265 mL), while the addition of the catalyst considerably enhanced the yield of permanent gases (from about 330 to 400 mL for cellulose conversion) (Table 1). This phenomenon was accompanied by a rapid growth of the efficiency of hydrogen production. Its amount increased from 1.3 mmol/g of cellulose in the case of the non-catalytic process to more than 10.0 mmol/g of cellulose for the reactions conducted in the presence of a catalyst. The highest H_2_ yield was achieved for Ni supported on MCF (15.9 mmol H_2_/g of cellulose). Production of other gaseous products were not much different when comparing yields obtained in the presence of the catalyst and yields obtained without a catalyst. The yield of CO and CO_2_ changed from 6.2 mmol CO/g of cellulose to 8.2 to 8.7 mmol CO/g of cellulose and from 1.2 mmol CO_2_/g of cellulose to 2.5 to 3.1 mmol CO_2_/g of cellulose for the non-catalytic and catalytic processes, respectively. The yield of generated CH_4_ remained on the similar level (about 1 mmol CH_4_/g of cellulose). As a result, the hydrogen contribution in the gaseous mixture increased from 14% (for non-catalytic process) to 57% (in the presence of the most active catalyst).

In the next step of the catalytic activity tests, the experiments with the use of real biomass sample (pine pulp) were performed. The results are summarized in Table 2. It can be seen that yields obtained using lignocellulosic feedstock are lower than yields obtained in cellulose conversion. Smaller H_2_ yields (associated with a more complex composition of the material subjected to thermal treatment) were observed in both the non-catalytic and catalytic reactions. The largest decrease in the catalyst activity was exhibited by the Ni/ZSM-5 sample, followed by Ni/ZrO_2_, while Ni/MCF yielded the highest H_2_ content among all tested catalysts.

### 3.2. Physicochemical Properties of the Catalysts

#### 3.2.1. Surface Area and Porosity

**Figure 1:**
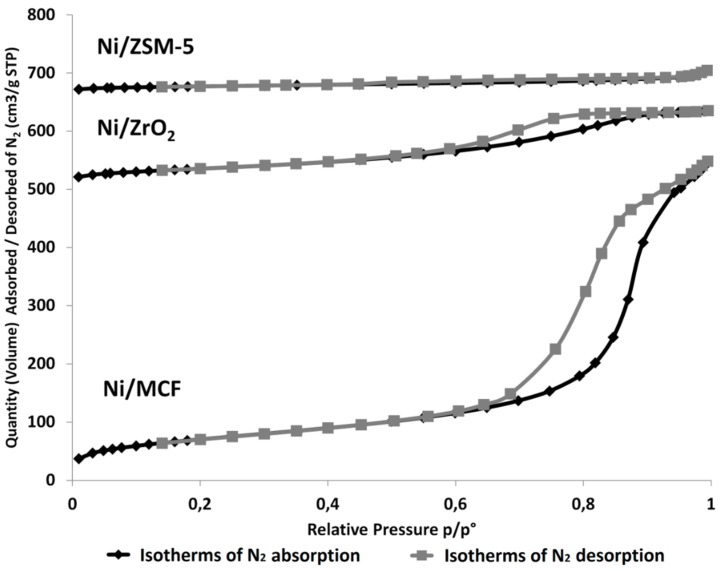
N_2_ adsorption/desorption isotherms obtained for Ni/MCF, Ni/ZrO_2_, and Ni/ZSM-5 catalysts.

Porosity characterization of the catalysts revealed that N_2_ adsorption/desorption isotherms obtained for Ni/MCF and Ni/ZrO_2_ samples (Figure 1) are very close to type IV (as defined by International Union of Pure and Applied Chemistry—IUPAC). In the case of Ni/MCF catalyst, a hysteresis loop related to type H1 is observed. This suggests that this material possesses mainly mesopores which shape is cylindrical with a constant cross-section. Ni/ZrO_2_ is characterized by H2 type of hysteresis loop implying the presence of mesopores with spherical shape, open ends, and numerous narrowings. On the other hand, the adsorption-desorption isotherm of Ni/ZSM-5 catalyst can be classified to type I with a hysteresis loop of type H4, characteristic for micropores in the shape of narrow slits [31].

Comparison of the results demonstrated that Ni/MCF and Ni/ZSM-5 exhibited the surface area of above 200 m^2^/g, while the surface area of Ni/ZrO_2_ was about two times lower. Ni/MCF catalyst was also characterized by a noticeably larger pore volume (0.85 mL/g) and pore radius (11.4 nm) in relation to other samples (Table 3). A similar trend was also observed in the literature [32].

#### 3.2.2. X-ray Diffraction (XRD) Analysis

**Figure 2:**
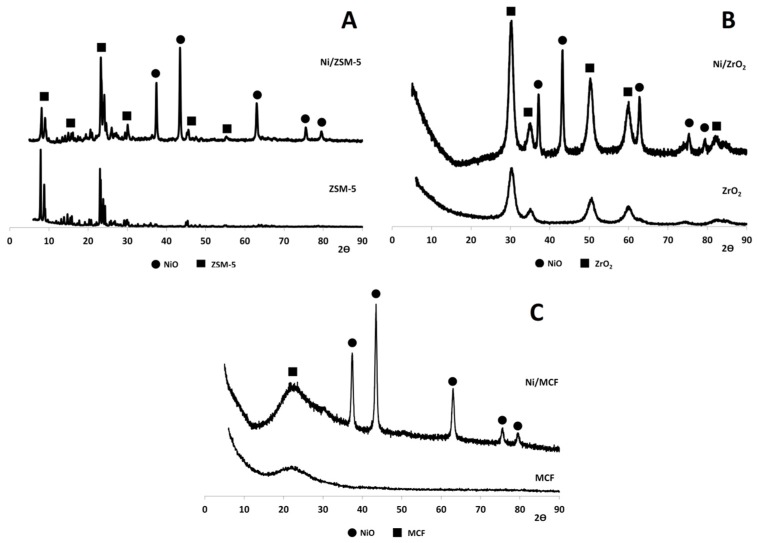
X-ray diffraction (XRD) diffractograms of: (**A**) ZSM-5 and Ni/ZSM-5, (**B**) ZrO_2_ and Ni/ZrO_2_, and (**C**) MCF and Ni/MCF.

The results of XRD measurements are shown in Figure 2. The diffraction peaks at *2θ* values of 37.2° (111), 43.2° (200), 62.8° (220), 75.3° (311), and 79.4° (222) were ascribed to the presence of nickel oxide phase [33]. Other signals originate from support components. *2θ* values of 11.0° (021), 14.0° (012), 15.9° (022), 18.2° (140), 23.1° (051), 24.0° (033), 24.6° (313), 29.5° (532), and 45.6° (100) were characteristic of ZSM-5 structure [34]. In the case of pure ZrO_2_ and Ni/ZrO_2_, the reflexes at 30.2° (101), 35.2° (002), 50.3° (200), and 60.2° (211) confirmed the presence of t-ZrO_2_ phase [35]. A broad signal at about 23° observed for MCF and Ni/MCF indicates the formation of an amorphous silica network of mesostructured cellular foam [32]. The structure of the supports did not change after introduction of the metal.

An estimation of NiO crystallite size performed using the Scherrer equation (Table 3) showed that the smallest average size of nickel oxide particles was observed in the case of the Ni/MCF sample (13 nm), while the size of NiO crystallites for Ni/ZrO_2_ and Ni/ZSM-5 was noticeably larger (22 nm and 31 nm, respectively).

#### 3.2.3. Temperature-Programmed Reduction (TPR)

**Figure 3:**
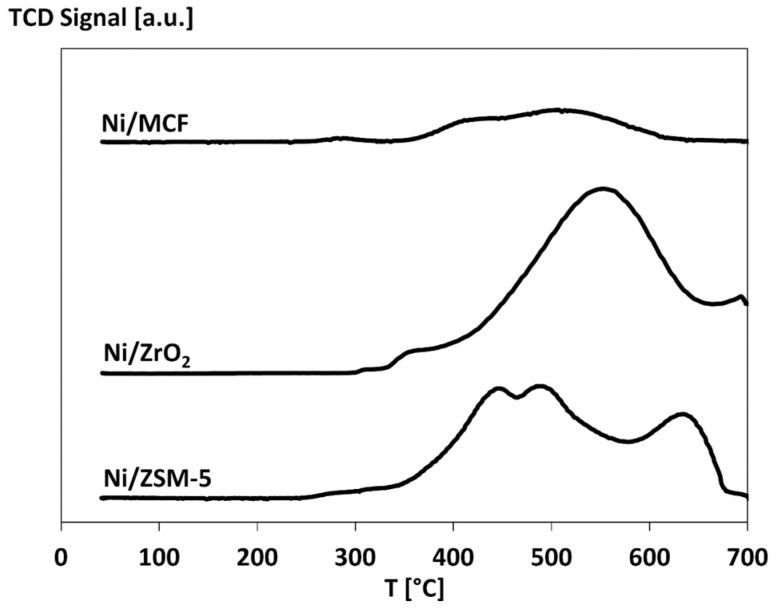
Temperature-programmed reduction (TPR) profiles of Ni catalysts.

TPR profiles presented in Figure 3 reveal that the reduction of nickel oxide introduced on the surface of various supports begins at the temperatures above 300 °C and completes below 700 °C. In the case of the Ni/ZSM-5 sample, the most intense consumption of hydrogen was observed between 350 °C and 550 °C, which corresponds to the reduction of NiO crystallites weakly interacting with the support [36]. The maximum of the signal corresponding to the second reduction range was noted at about 650 °C. The reduction at this temperature can be ascribed to stronger interactions between Ni active phase and ZSM5 support [37,38].

The reduction of nickel oxide supported on zirconia occurs in one broad temperature range with the maximum hydrogen consumption above 550 °C. The highest contribution of NiO species undergoing reduction at elevated temperatures can be ascribed to the highest strength of the interactions between the support and the active phase among all catalysts [39].

The TPR profile recorded for Ni/MCF catalyst has a broad reduction temperature range with two poorly separated maxima, observed at about 400 °C and 500 °C. In this case, the hydrogen consumption occurs mainly in the lowest temperature region. It corresponds to the reduction of NiO having a weak interaction with mesostructured cellular foam or to the reduction of small crystallites of the active phase [40].

#### 3.2.4. Temperature-Programmed Desorption (TPD) of Ammonia

**Figure 4:**
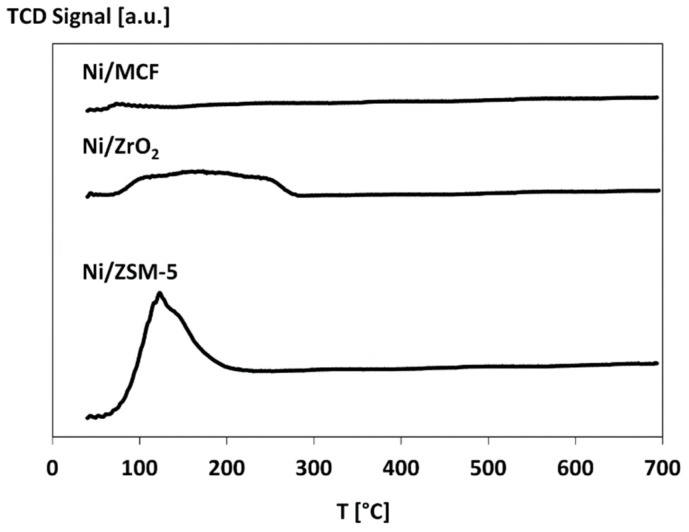
Temperature-programmed desorption (TPD)-NH_3_ profiles of Ni catalysts.

The results of temperature-programmed desorption (TPD)-NH_3_ measurements (Table 3) reveal that Ni/ZSM-5 catalyst possesses considerably higher surface acidity (905 mmol/NH_3_/g) than other samples (187 and 56 mmol/NH_3_/g for Ni/ZrO_2_ and Ni/MCF, respectively). TPD profiles for ammonia (Figure 4) demonstrate that desorption of ammonia generally occurs at temperatures not higher than 300 °C. However, in the case of the most acidic Ni/ZSM-5 catalyst, this process was observed only between 80 °C and 200 °C. High acidity of catalysts supported on ZSM-5 in comparison to MCF [41] and ZrO_2_ [16] has been previously observed in Reference [42].

#### 3.2.5. Scanning Electron Microscopy (SEM)

**Figure 5:**
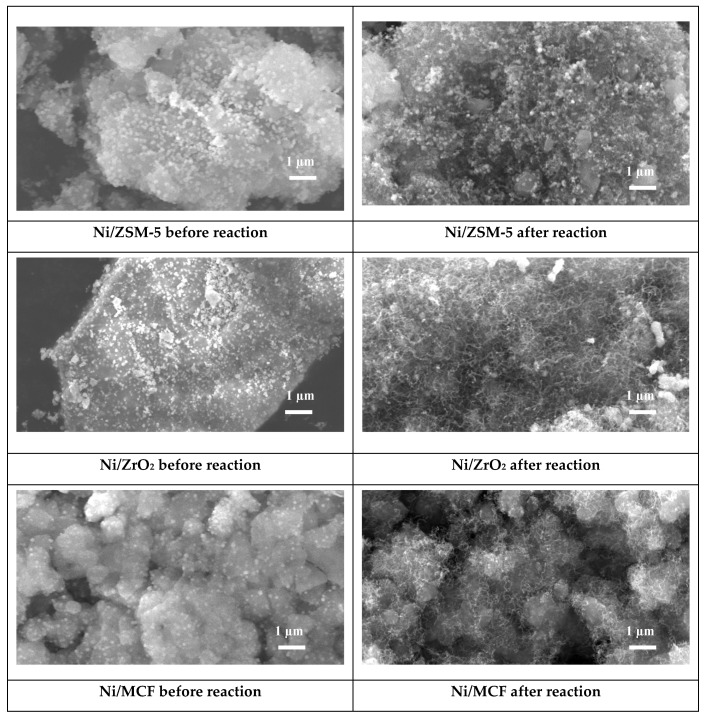
Scanning electron microscopy (SEM) images of the catalyst before and after cellulose decomposition process (magnification 10,000×).

Figure 5 shows SEM images of the catalysts obtained before and after reaction.

SEM images collected from the surface of the catalysts demonstrated differences in their morphology. In the case of the Ni/ZrO_2_ sample, large crystallites of support with the most compact structure were observed. On the other hand, for Ni/ZSM-5 and Ni/MCF catalysts, agglomerates of support microcrystallites were noted. Their size was the smallest for the system containing MCF. This observation is in agreement with the results of surface area presented in Table 3 and literature reports [32].

An analysis of the results of EDX analysis (Table 4) confirmed introduction of nickel into the structure of the support. However, its surface content varied (from 10.7% to 20.5%) depending on the type of support used. This trend is opposite to the change in the mean size of NiO crystallites calculated on the basis of XRD results, which showed that nickel oxide agglomerates were the largest for Ni/ZSM-5 and the smallest in the case of Ni/MCF (Table 4). It can be suggested that the smallest particles of an active phase can efficiently be deposited inside pores of MCF since MCF pores possess the largest diameter among tested samples and the largest pore volume. On the other hand, ZSM-5 possesses the smallest pore size and large NiO particles cannot easily penetrate them. That is why nickel remains mainly on the surface of this material. SEM images recorded for the catalysts after cellulose conversion (Figure 5) show the formation of carbon deposits. Observed carbonaceous deposits are mainly in the form of carbon whiskers. A similar shape of coke was observed after high-temperature conversion of lignocellulosic feedstock [43].

#### 3.2.6. Thermogravimetric Analysis (TGA)

Thermogravimetric experiments were conducted in order to evaluate the amount and reactivity of carbon deposited on the surface of the catalysts. The experiments were performed for the samples used in both cellulose and pine decomposition. Quantitative analysis of TG curves (Table 5) revealed that in the case of cellulose conversion, the highest amount of deposited carbon was observed for Ni/ZSM-5 and Ni/MCF catalysts (15.3 and 12.5 wt.%, respectively), while in the case of Ni/ZrO_2_, carbon deposition was about two times lower (6.6 wt.%). A different trend was observed in the case of pine decomposition. In this case, the amount of carbon on the surface of Ni/MCF and Ni/ZrO_2_ remained at a high and moderate levels, respectively (15.5 and 6.1 wt.%). However, the carbon content obtained on Ni/ZSM-5 was considerably lower (3.3 wt.%). This phenomenon is related with the fastest drop in the activity of the Ni/ZSM-5 in the presence of lignin, which will be discussed later.

**Figure 6:**
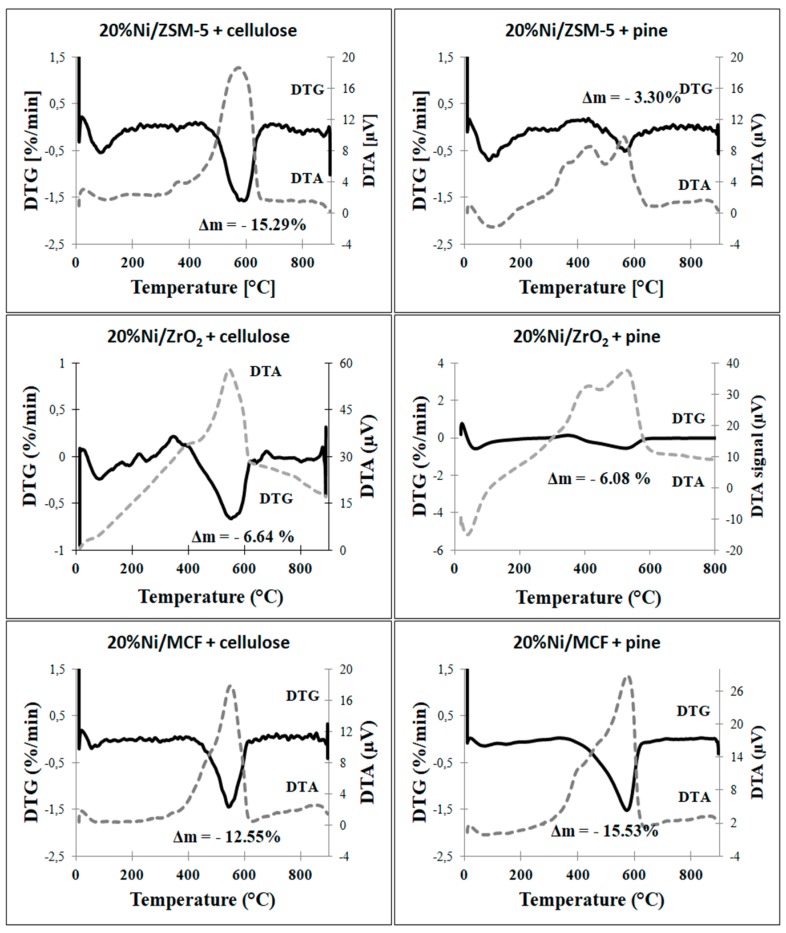
TGA-DTA curves of the catalysts.

Qualitative analysis of TG results (Figure 6) demonstrated that in all cases, the main part of carbon deposit was removed in the temperature range between 400 °C and 600 °C. However, the maximal weight loss was observed between 550 °C and 600 °C. Additionally, it is worth noting that the maximum slightly shifted towards higher temperatures for the catalysts used for pine conversion in comparison to the samples used for cellulose decomposition. This phenomenon may be related to the formation of more graphitic carbon in the presence of lignin [44].

## 4. Discussion

High-temperature conversion of lignocellulosic biomass is a complex process consisting of several steps. In the first of them, thermal decomposition of individual biomass components takes place. Depolymerization, elimination, and dehydration reactions result in the formation of a large number of oxygenates, which are subjected to cracking, reforming, decarboxylation, decarbonylation, dehydratation, and oligomerization in the subsequent steps [45]. These reactions lead to the production of a wide range of secondary products, among which, hydrogen and other light molecules can also be distinguished [46]. The main role of the catalyst is to facilitate cracking and reforming reactions, which in turn, increase an efficiency of the formation of gaseous products and facilitate production of molecular hydrogen, limiting contribution of undesirable substances [47].

The experiments presented here revealed considerable differences in the efficiency of Ni catalyst supported on ZSM-5, ZrO_2_ and MCF in the high-temperature conversion of cellulose and real biomass samples towards hydrogen-rich gas. It is related to different properties of these materials.

It is known that higher surface acidity of the support favors cracking of large reaction intermediates to smaller molecules [48]. Taking that into account, it would be expected that contribution of gaseous products formed in the presence of Ni/ZSM-5 should be the largest. However, strong acid sites undergo fast deactivation by the adsorption of carbonaceous species [49]. Therefore, an activity of Ni/ZSM-5 is lower than that observed for other catalysts. Moreover, an application of zeolite leads to the formation of a slightly larger amount of methane.

A comparison of the structural parameters of the samples revealed that in spite of the similar surface areas of Ni/ZSM-5 and Ni/MCF, the latter catalyst exhibits higher activity in the production of hydrogen-rich gas from lignocellulosic feedstock. This phenomenon is likely due to noticeably larger pore radius and pore volume enabling more efficient contact between an active phase of the catalyst and large primary products formed in the first step of the high-temperature treatment of lignocellulosic feedstock. [50]. Due to this fact, more efficient formation of gaseous products is favored [31]. Our results demonstrate that pore diameter and their volume contribute to the increase in hydrogen yield to a greater extent than the acidity of the support.

The size of active phase particles and their location are another two factors influencing catalyst activity. The presence of smaller NiO crystallites enhances the reaction yield due to the formation of a larger number of active sites on the catalyst surface. Moreover, small particles can be located inside the pores of the support, not only on its surface. This phenomenon was confirmed for the most active Ni/MCF catalyst by both XRD and EDX measurements. On the other hand, smaller nickel species may be subjected to more intense coking leading to faster deactivation of the catalyst [51].

TPR experiments revealed that the interactions between an active phase and the support in the case of the most active Ni/MCF catalyst are moderate. Weak interactions may result in sintering of nickel crystallites, while too-strong interactions may lead to the difficulty of nickel oxide reduction during the reaction and overall changes in the properties of the catalyst [52].

A comparison of the coking intensity of the catalysts during the conversion of cellulose shows that the largest amount of carbon was deposited on the surface of Ni/ZSM-5. This is due to the highest acidity of the material [42]. On the other hand, the coke content in the case of Ni/ZrO_2_ was considerably lower. Literature data shows that ZrO_2_ can adsorb water molecules and facilitate their further decomposition, which leads to the formation of hydroxyl groups taking part in the removal (gasification) of carbonaceous species deposited during the reaction [53]. An analysis of the content of carbon deposited on the surface of the most active Ni/MCF demonstrated that the catalyst is not particularly resistant to coking. However, a presence of more filamentous coke and larger pores prevent it from blocking Ni active centers [31]. Therefore, even in the case of pine conversion, this catalyst allows to achieve the highest hydrogen yield.

A decrease in the amount of deposited carbon observed for Ni/ZSM-5 during decomposition of the real biomass sample is most likely due to fast blockage of active centers on the surface of the catalyst by deposited coke. The plugging of the active centers results in the highest decrease in the catalytic activity in comparison to the cellulose conversion process. This can be attributed to the presence of lignin in the composition of pine, which enhances coking and formation of large reaction intermediates (tar) [54].

To summarize, an application of MCF as a support of Ni catalyst for the high-temperature conversion of cellulose allows for the production of considerably higher amounts of H_2_ (15.9 mmol H_2_/g cellulose) in comparison with the amount of H_2_ produced by Ni supported on a commercial monoxide silica, alumina, or ceria (about 10 mmol H_2_/g cellulose) [18,55]. The efficiency of Ni/MCF was also higher than that observed for Ni/ZrO_2_ and Co/SBA-15 or Ni-Co/SBA-15 (13.0–13.5 mmol H_2_/g cellulose) [20,56,57] and similar to the performance of Ni/CaAlO_x_ catalyst (13.0–15.6 mmol H_2_/g biomass depending on the value of Ca/Al ratio) [58].

## 5. Conclusions

The results obtained in this study show a strong influence of porosity and acidity of the support, crystallites size of the active phase, and strength of their interaction with the support on the Ni catalyst performance in the production of hydrogen-rich gas by high-temperature conversion of lignocellulosic feedstock. Ni/MCF catalyst, characterized by large pore size and pore volume, low acidity, small NiO crystallite size and moderate interaction with the active phase, were the most efficient in the process. The high acidity of ZSM-5 did not lead to an increase in the number of gaseous products in the reaction mixture, but rather resulted in the reduction of catalyst activity. A comparison of physicochemical properties of the catalysts suggests that large pore size and a better contact between the active phase and reaction intermediates have a greater impact on the activity of the catalysts towards the formation of light molecules (including hydrogen) than the acidity of the support.

## Figures and Tables

**Table 1:** Gaseous products formed in high-temperature conversion of cellulose.

Catalyst	Gas Volume (mL)	H_2_ (mmol/g)	CO_2_ (mmol/g)	CH_4_ (mmol/g)	CO (mmol/g)
Without Catalyst	220	1.3	1.2	0.9	6.2
ZSM-5	265	1.2	0.9	1.0	8.5
ZrO_2_	245	1.3	0.8	1.0	7.1
MCF	230	1.4	0.8	0.9	6.4
Ni/ZSM-5	335	10.0	2.9	1.1	8.6
Ni/ZrO_2_	382	13.0	3.1	0.9	8.2
Ni/MCF	407	15.9	2.5	0.9	8.7

**Table 2:** Gaseous products formed in high-temperature conversion of pine pulp.

Catalyst	Gas Volume (mL)	H_2_ (mmol/g)	CO_2_ (mmol/g)	CH_4_ (mmol/g)	CO (mmol/g)
Without Catalyst	194	0.8	0.7	0.9	7.7
ZSM-5	215	0.7	0.5	1.0	8.0
ZrO_2_	210	1.0	0.7	1.0	7.4
MCF	190	1.1	0.7	0.9	6.1
Ni/ZSM-5	310	4.4	1.1	1.4	9.4
Ni/ZrO_2_	337	7.8	1.4	1.1	8.1
Ni/MCF	363	11.0	1.8	1.0	8.8

**Table 3:** Selected physicochemical properties of the supports and Ni catalysts.

Catalyst	BET Surface Area (m^2^/g)	Pore Volume (mL^3^/g)	Pore Radius (nm)	Acidity (mmol NH_3_/g)	NiO Crystallite Size (nm)
ZSM-5	298	0.06	2.1	1120	-
ZrO_2_	217	0.37	2.7	610	-
MCF	631	2.40	13.7	97	-
Ni/ZSM-5	237	0.06	3.1	905	31
Ni/ZrO_2_	127	0.21	2.7	187	22
Ni/MCF	252	0.85	11.4	56	13

**Table 4:** Concentration of main elements calculated on the basis of scanning electron microscopy-energy-dispersive X-ray (SEM-EDX) measurements (at.%).

Element	Ni/ZSM-5	Ni/ZrO_2_	Ni/MCF
O	27.7	8.7	30.9
Al	3.4	-	-
Si	48.0	-	57.7
Ni	20.5	15.2	10.7
Zr	-	67.5	-

**Table 5:** Content of carbon deposit calculated on the basis of thermogravimetric analysis/differential thermal analysis (TGA/DTA) results.

Catalyst	Carbon Deposit (%)	
	Cellulose	Pine
Ni/ZSM-5	15.3	3.3
Ni/ZrO_2_	6.6	6.1
Ni/MCF	12.5	15.5

## References

[1] Huang Y.F., Chiueh P.T., Kuan W.H., Lo S.L. (2018). Product distribution and heating performance of lignocellulosic biomass pyrolysis using microwave heating. Energy Procedia.

[2] Mahmood H., Moniruzzaman M., Iqbal T., Khan M.J. (2019). Recent advances in the pretreatment of lignocellulosic biomass for biofuels and value-added products. Curr. Opin. Green Sustain. Chem..

[3] Kucharska K., Hołowacz I., Konopacka-Łyskawa D., Rybarczyk P., Kamiński M. (2018). Key issues in modeling and optimization of lignocellulosic biomass fermentative conversion to gaseous biofuels. Renew. Energy.

[4] Persson H., Yang W. (2019). Catalytic pyrolysis of demineralized lignocellulosic biomass. Fuel.

[5] Chen X., Che Q., Li S., Liu Z., Yang H., Chen Y., Wang X., Shao J., Chen H. (2019). Recent developments in lignocellulosic biomass catalytic fast pyrolysis: Strategies for the optimization of bio-oil quality and yield. Fuel Process. Technol..

[6] Jing Y., Guo Y., Xia Q., Liu X., Wang Y. (2019). Catalytic production of value-added chemicals and liquid fuels from lignocellulosic biomass. Chem.

[7] Gai C., Zhu N., Hoekman S.K., Liu Z., Jiao W., Peng N. (2019). Highly dispersed nickel nanoparticles supported on hydrochar for hydrogen-rich syngas production from catalytic reforming of biomass. Energy Convers. Manag..

[8] Dong Q., Zhang S., Li H., Li X., Wang Z. (2019). Catalytic cracking of biomass tar together with syngas production over red brick powder-supported nickel catalysts. Fuel Process. Technol..

[9] Grams J., Ruppert A. (2017). Development of heterogeneous catalysts for thermo-chemical conversion of lignocellulosic biomass. Energies.

[10] Moghtaderi B. (2007). Effects of controlling parameters on production of hydrogen by catalytic steam gasification of biomass at low temperatures. Fuel.

[11] Li J., Liu J., Liao S., Yan R. (2010). Hydrogen-rich gas production by air-steam gasification of rice husk using supported nano-NiO/γ-Al_2_O_3_ catalyst. Int. J. Hydrogen Energy.

[12] Chen D., He L. (2011). Towards an efficient hydrogen production from biomass: A review of processes and materials. ChemCatChem.

[13] Lu Y., Li S., Guo L., Zhang X. (2010). Hydrogen production by biomass gasification in supercritical water over Ni/γ-Al_2_O_3_ and Ni/CeO_2_-γ-Al_2_O_3_ catalysts. Int. J. Hydrogen Energy.

[14] Tomishige K., Kimura T., Nishikawa J., Miyazawa T., Kunimori K. (2007). Promoting effect of the interaction between Ni and CeO_2_ on steam gasification of biomass. Catal. Commun..

[15] Park H.J., Park S.H., Sohn J.M., Park J., Jeon J.K., Kim S.S., Park Y.K. (2010). Steam reforming of biomass gasification tar using benzene as a model compound over various Ni supported metal oxide catalysts. Bioresour. Technol..

[16] Grams J., Niewiadomski M., Ryczkowski R., Ruppert A.M., Kwapiński W. (2016). Activity and characterization of Ni catalyst supported on CeO_2_-ZrO_2_ for thermo-chemical conversion of cellulose. Int. J. Hydrogen Energy.

[17] Kong M., Fei J., Wang S., Lu W., Zheng X. (2011). Influence of supports on catalytic behavior of nickel catalysts in carbon dioxide reforming of toluene as a model compound of tar from biomass gasification. Bioresour. Technol..

[18] Matras J., Niewiadomski M., Ruppert A., Grams J. (2012). Activity of Ni catalysts for hydrogen production via biomass pyrolysis. Kinet. Catal..

[19] Silveira E.B., Rabelo-Neto R.C., Noronha F.B. (2017). Steam reforming of toluene, methane and mixtures over Ni/ZrO_2_ catalysts. Catal. Today.

[20] Ruppert A.M., Niewiadomski M., Grams J., Kwapiński W. (2014). Optimization of Ni/ZrO_2_ catalytic performance in thermochemical cellulose conversion for enhanced hydrogen production. Appl. Catal. B Environ..

[21] Inaba M., Murata K., Saito M., Takahara I. (2006). Hydrogen production by gasification of cellulose over Ni catalysts supported on zeolites. Energy Fuels.

[22] French R., Czernik S. (2010). Catalytic pyrolysis of biomass for biofuels production. Fuel Process. Technol..

[23] Melligan F., Hayes M.H.B., Kwapinski W., Leahy J.J. (2012). Hydro-pyrolysis of biomass and online catalytic vapor upgrading with Ni-ZSM-5 and Ni-MCM-41. Energy Fuels.

[24] Karnjanakom S., Guan G., Asep B., Hao X., Kongparakul S., Samart C., Abudula A. (2016). Catalytic upgrading of bio-oil over Cu/MCM-41 and Cu/KIT-6 prepared by β-cyclodextrin-assisted coimpregnation method. J. Phys. Chem. C.

[25] Grams J., Potrzebowska N., Goscianska J., Michalkiewicz B., Ruppert A.M. (2016). Mesoporous silicas as supports for Ni catalyst used in cellulose conversion to hydrogen rich gas. Int. J. Hydrogen Energy.

[26] Che Q., Yang M., Wang X., Yang Q., Chen Y., Chen X., Chen W., Hu J., Zeng K., Yang H. (2019). Preparation of mesoporous ZSM-5 catalysts using green templates and their performance in biomass catalytic pyrolysis. Bioresour. Technol..

[27] Wei L., Zhao Y., Zhang Y., Liu C., Hong J., Xiong H., Li J. (2016). Fischer-Tropsch synthesis over a 3D foamed MCF silica support: Toward a more open porous network of cobalt catalysts. J. Catal..

[28] Santamaria L., Arregi A., Alvarez J., Artetxe M., Amutio M., Lopez G., Bilbao J., Olazar M. (2018). Performance of a Ni/ZrO_2_ catalyst in the steam reforming of the volatiles derived from biomass pyrolysis. J. Anal. Appl. Pyrolysis.

[29] Schmidt-Winkel P., Lukens W.W., Yang P., Margolese D.I., Lettow J.S., Ying J.Y., Stucky G.D. (2000). Microemulsion templating of siliceous mesostructured cellular foams with well-defined ultralarge mesopores. Chem. Mater..

[30] Schmidt-Winkel P., Lukens W.W., Zhao D., Yang P., Chmelka B.F., Stucky G.D. (1999). Mesocellular siliceous foams with uniformly sized cells and windows. J. Am. Chem. Soc..

[31] Widyaningrum R.N., Church T.L., Zhao M., Harris A.T. (2012). Mesocellular-foam-silica-supported Ni catalyst: Effect of pore size on H_2_ production from cellulose pyrolysis. Int. J. Hydrogen Energy.

[32] Hermida L., Abdullah A.Z., Mohamed A.R., A. Mendez-Vilas A. (2013). Nickel functionalized mesostructured cellular foam ( MCF ) silica as a catalyst for solventless deoxygenation of palmitic acid to produce diesel-like hydrocarbons. Materials and Processes for Energy: Communicating Current Research and Technological Developments.

[33] El-Kemary M., Nagy N., El-Mehasseb I. (2013). Nickel oxide nanoparticles: Synthesis and spectral studies of interactions with glucose. Mater. Sci. Semicond. Process..

[34] Possato L.G., Acevedo M.D., Padró C.L., Briois V., Passos A.R., Pulcinelli S.H., Santilli C.V., Martins L. (2018). Activation of Mo and V oxides supported on ZSM-5 zeolite catalysts followed by in situ XAS and XRD and their uses in oxydehydration of glycerol. Mol. Catal..

[35] Davar F., Hassankhani A., Loghman-Estarki M.R. (2013). Controllable synthesis of metastable tetragonal zirconia nanocrystals using citric acid assisted sol-gel method. Ceram. Int..

[36] Xu Z., Li Y., Zhang J., Chang L., Zhou R., Duan Z. (2001). Bound-state Ni species - a superior form in Ni-based catalyst for CH_4_/CO_2_ reforming. Appl. Catal. A Gen..

[37] Iriondo A., Cambra J.F., Güemez M.B., Barrio V.L., Requies J., Sánchez-Sánchez M.C., Navarro R.M. (2012). Effect of ZrO_2_ addition on Ni/Al_2_O_3_ catalyst to produce H_2_ from glycerol. Int. J. Hydrogen Energy.

[38] Rzeznicka I.I., Góralski J., Paryjczak T. (2001). Role of support on the reducibility of NiO. Chem. Environ. Res..

[39] Maia T.A., Assaf E.M. (2014). Catalytic features of Ni supported on CeO_2_-ZrO_2_ solid solution in the steam reforming of glycerol for syngas production. RSC Adv..

[40] Amin R., Liu B., Ullah S., Biao H.Z. (2017). Study of coking and catalyst stability over CaO promoted Ni-based MCF synthesized by different methods for CH_4_/CO_2_ reforming reaction. Int. J. Hydrogen Energy.

[41] Sobczak I., Wolski Ł. (2015). Au-Cu on Nb_2_O_5_ and Nb/MCF supports - Surface properties and catalytic activity in glycerol and methanol oxidation. Catal. Today.

[42] Veses A., Puértolas B., Callén M.S., García T. (2015). Catalytic upgrading of biomass derived pyrolysis vapors over metal-loaded ZSM-5 zeolites: Effect of different metal cations on the bio-oil final properties. Microporous Mesoporous Mater..

[43] Ryczkowski R., Ruppert A.M., Przybysz P., Chałupka K., Grams J. (2017). Hydrogen production from biomass woodchips using Ni/CaO–ZrO_2_ catalysts. React. Kinet. Mech. Catal..

[44] Zhang Z., Ou Z., Qin C., Ran J., Wu C. (2019). Roles of alkali/alkaline earth metals in steam reforming of biomass tar for hydrogen production over perovskite supported Ni catalysts. Fuel.

[45] Navarro R.M., Peña M.A., Fierro J.L.G. (2007). Hydrogen production reactions from carbon feedstocks: Fossil fuels and biomass. Chem. Rev..

[46] Minowa T., Ogi T. (1998). Hydrogen production from cellulose using a reduced nickel catalyst. Catal. Today.

[47] Bulushev D.A., Ross J.R.H. (2011). Catalysis for conversion of biomass to fuels via pyrolysis and gasification: A review. Catal. Today.

[48] Tanksale A., Beltramini J.N., Lu G.Q.M. (2010). A review of catalytic hydrogen production processes from biomass. Renew. Sustain. Energy Rev..

[49] Liu N., Xie H., Cao H., Shi L., Meng X. (2019). Multi-technique characterization of recycled acetylene carbonylation catalyst CuY: Deactivation and coke analysis. Fuel.

[50] Gao S., Xu S., Wei Y., Qiao Q., Xu Z., Wu X., Zhang M., He Y., Xu S., Liu Z. (2018). Insight into the deactivation mode of methanol-to-olefins conversion over SAPO-34: Coke, diffusion, and acidic site accessibility. J. Catal..

[51] Titus J., Roussière T., Wasserschaff G., Schunk S., Milanov A., Schwab E., Wagner G., Oeckler O., Gläser R. (2016). Dry reforming of methane with carbon dioxide over NiO-MgO-ZrO_2_. Catal. Today.

[52] Zhang B., Zhang L., Yang Z., He Z. (2017). An experiment study of biomass steam gasification over NiO/Dolomite for hydrogen-rich gas production. Int. J. Hydrogen Energy.

[53] Arslan A., Gunduz S., Dogu T. (2014). Steam reforming of ethanol with zirconia incorporated mesoporous silicate supported catalysts. Int. J. Hydrogen Energy.

[54] Muley P.D., Henkel C., Abdollahi K.K., Marculescu C., Boldor D. (2016). A critical comparison of pyrolysis of cellulose, lignin, and pine sawdust using an induction heating reactor. Energy Convers. Manag..

[55] Grams J., Goscianska J., Potrzebowska N., Ryczkowski R., Michalkiewicz B., Ruppert A.M. (2017). Impact of Zr incorporation into the Ni/AlSBA-15 catalyst on its activity in cellulose conversion to hydrogen-rich gas. Energy Fuels.

[56] Zhao M., Florin N.H., Harris A.T. (2010). Mesoporous supported cobalt catalysts for enhanced hydrogen production during cellulose decomposition. Appl. Catal. B.

[57] Zhao M., Church T.L., Harris A.T. (2011). SBA-15 supported Ni-Co bimetallic catalysts for enhanced hydrogen production during cellulose decomposition. Appl. Catal. B.

[58] Chen F., Wu C., Dong L., Vassallo A., Williams P.T., Huang J. (2016). Characteristics and catalytic properties of Ni/CaAlOx catalyst for hydrogen-enriched syngas production from pyrolysis-steam reforming of biomass sawdust. Appl. Catal. B.

